# Coexistence process and driving factors of arbuscular mycorrhizal fungi in urban green soil under heavy metal stress

**DOI:** 10.1128/aem.00171-26

**Published:** 2026-03-10

**Authors:** Haopu Li, Yazhou Feng, Zhonghu Geng, Mengyao Zhang, Yiming Cui, Yizhen Shao, Yun Chen, Zhiliang Yuan

**Affiliations:** 1 College of Life Science, Henan Agricultural University 70573, Zhengzhou, China; Colorado School of Mines, Golden, Colorado, USA

**Keywords:** heavy metal, urban green spaces, AMF, community assembly process

## Abstract

Urban green spaces in rapidly developing cities face severe heavy metal (HM) pollution, yet how this pollution affects arbuscular mycorrhizal fungal (AMF) community assembly remains poorly understood. This study involved the collection of 60 soil samples from green spaces throughout Zhengzhou, China. Soil edaphic factors and levels of five HMs were measured, alongside the sequencing of AMF communities. Our results indicated that all HMs exceeded Henan soil background levels, with pollution levels increasing with greater distance from the urban center. Furthermore, deterministic processes governed the assembly of specialist AMF communities, while stochastic processes dominated in generalists. Iron (Fe) and zinc (Zn) served as key drivers of the AMF community structure. These findings highlight the significant role of HM pollution in shaping AMF community assembly processes in urban green spaces. By enhancing our understanding of how HM pollution affects AMF ecological dynamics, strategies can be informed for maintaining and improving ecosystem functionality in urban green areas.

**IMPORTANCE:**

This study highlights the growing threat of heavy metal pollution in urban green spaces, which poses risks to soil health and ecosystem stability. As cities rapidly expand, increased pollution in green areas can disrupt microbial communities crucial for soil function. Our research shows that heavy metal contamination is increasingly affecting arbuscular mycorrhizal fungi vital for plant growth and soil quality. This shift in fungal communities is driven by rising pollution levels, with significant impacts on urban ecosystems. Understanding how these pollution-driven changes shape microbial communities is crucial for managing urban soil health. These insights are especially timely, as urbanization accelerates worldwide, presenting new challenges for environmental protection. The findings are relevant for urban planning, offering strategies to mitigate the effects of pollution and maintain ecosystem functions in green spaces. This research contributes to the broader goal of preserving sustainable urban environments in the face of escalating environmental stress.

## INTRODUCTION

Heavy metal (HM) pollution has become a globally concerning environmental issue in recent decades. Urban green spaces are ecosystems characterized by diverse pollution sources of green land soil. Within such spaces, the degree of HM pollution tends to be serious (1). High concentrations of HMs have been shown to have a toxic effect on microorganisms, leading to alterations in microbial community structure (2, 3). Metal(loid)s can build up in microorganisms, even at low concentrations, leading to cell structure damage, metabolic disruptions, and the denaturation of proteins, DNA, and other molecules. Therefore, HMs are considered one of the major abiotic stresses affecting soil microbial populations and community structure, especially arbuscular mycorrhizal fungi (AMF) (4, 5).

More than 80% of vascular plants have symbiotic interactions with AMF, which improves their absorption of nutrients and water (6). AMF also improve soil properties and act as barriers by immobilizing HMs within plant roots through phytostabilization (7, 8). Research has shown that plants associated with AMF exhibit superior growth performance in HM-contaminated soils compared to non-mycorrhizal plants, with greater HM tolerance and biomass accumulation capacity (9). The specific concentration and form of HM pollutants significantly affect the diversity of AM fungi through several factors, such as mycelial biomass, infectivity reduction, and abundance (10). Excessive HM levels are toxic to most AMF, allowing only those with high tolerance to survive, thus leading to a reduction in AMF diversity (11). Thus, investigating the shifts in AMF community diversity in urban green space soils affected by heavy metals (HMs) and further examining the mechanisms by which AMF respond to HM pollution are crucial for managing urban green space ecosystems.

One of the main goals of microbial ecology is to quantify the proportional contributions of deterministic and stochastic processes to the formation of microbial communities (12). Migration and dispersal play a significant role in shaping stochastic processes (13), while deterministic processes are driven by species traits, biological interactions, and environmental filtering (14). The assembly mechanism of microbial communities under HM stress plays a crucial role in shaping biodiversity and community composition (15). Microbial communities are subject to strong selective pressure exerted by HMs (16). Increased biohomogenization results from the environmental selection pressures imposed by HM, which lessens the impact of random processes in the assembly of AMF communities (13). It is still unclear how AMF communities assemble in HM-contaminated urban green space soils, especially when it comes to specialists and generalists based on the width of their niches. Understanding the fundamental mechanisms that maintain the AMF community assembly is essential for grasping how microbial communities respond to and interact with HM pollution in these ecosystems.

Zhengzhou, as one of the fastest-growing cities in the Central Plains of China, has witnessed continuous economic and transportation development in recent years while also facing major challenges in environmental protection. In the current study, we studied the distribution patterns of AMF communities and soil pollutants across 60 green space sites along the urban-rural gradient. Therefore, our objectives are to (i) assess the changes and levels of soil conditions and HMs along different levels of urbanization, (ii) analyze how soil AMF communities react to this gradient, and (iii) look into how AMF communities assemble and what influences them in urban green soils when heavy metal contamination is present.

## MATERIALS AND METHODS

### Study area and classification

With a total area of 7,567.18 km², including an urban area of 1,078.07 km² (34°16′–34°58′ N, 112°42′–114°13′ E), Zhengzhou City in central China was the site of this study. Zhengzhou City is situated in the warm temperate and subtropical transition zones, with a typical warm temperate continental monsoon climate. The average temperature is 2.42°C in January and 25.9°C in August, while the city receives an average annual rainfall of 690 mm.

The vegetation in Zhengzhou City is classified as warm temperate deciduous broad-leaved forest, featuring diverse plant resources. Common green space plants within the sample plots include *Platanus*, *Firmiana simplex*, *Ginkgo biloba*, *Ligustrum lucidum*, and *Styphnolobium japonicum*. The total area of green spaces in the urban built-up areas is 233.51 million sqm; the per capita public green space is 13.6 km^2^; and the green coverage rate is 45%.

### Sample collection

**Fig 1:**
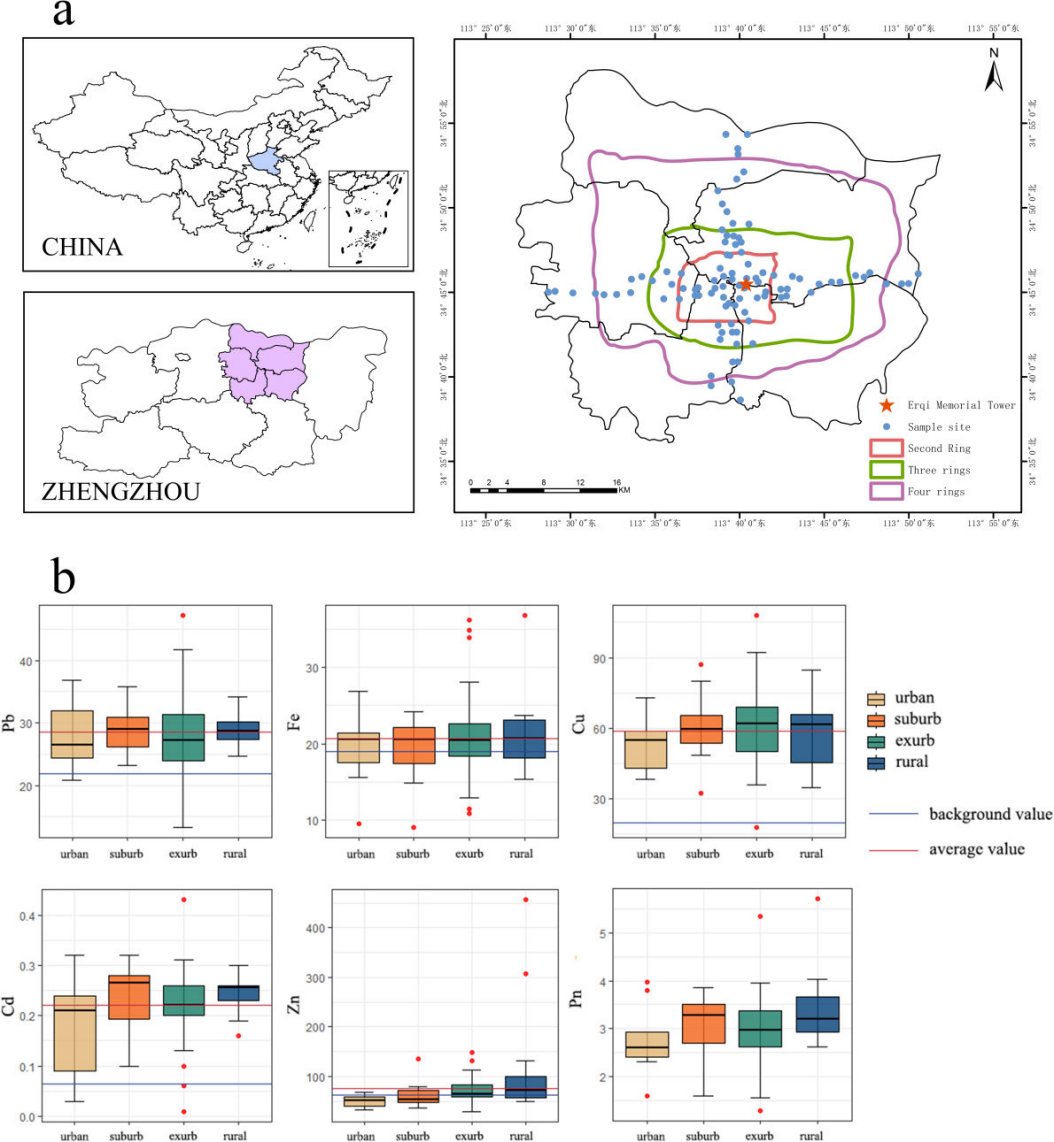
The locations of the 60 soil samples collected in Zhengzhou (**a**) and the levels of five heavy metals (HMs) and the Pn (Nemerow composite pollution index) across urban categories (**b**) are shown. The 60 sites are categorized as urban (13 sites), suburban (14 sites), exurban (21 sites), and rural (12 sites). The maps were created using ArcGIS, with the base map sourced from the official China Standard Map Service under Map Review Number GS(2019)1822.

Starting from the city center of Zhengzhou (Erqi District), a 3 km-wide survey transect was set up along the four directions of east, west, south, and north. Within the survey transect, establish 60 sample plots (10 m × 10 m) in urban green spaces, with each plot separated by at least 1 km. We categorized 60 sites in Zhengzhou (China) into four groups based on urban development rings: 13 sites in urban areas, 14 in suburban areas, 21 in exurban areas, and 12 in rural areas (Fig. 1). The area inside the Second Ring was classified as “urban”; the zone between the second and third rings was “suburban”; the region between the third and fourth rings was “exurban”; and the area beyond the Fourth Ring was “rural.” We calculated the distance of each site from the urban center using Euclidean distance in ArcGIS 10.2 based on the projected coordinate system (WGS 1984 UTM zone 51 N).

Within each 10 m × 10 m sample plot, select three sampling points to collect soil from the plant root zone. At each sampling point, after clearing surface litter and gravel, collect plant root soil from a depth of 0 to 20 cm. The soil from three points was sieved using a 2 mm mesh and combined to create a composite sample for each quadrat. The mixed samples were divided into two halves, one of which was kept at −80°C for Illumina sequencing and the other at 4°C for the investigation of soil physicochemical properties.

### Soil edaphic conditions and HM measurements

Soil physiochemical properties include soil organic matter (SOM), available phosphorus (AP), soil moisture content (SMC), available potassium (AK), soil pH, and total nitrogen (TN). We referred to a previous study for the determination of these indicators. After digesting the samples with a solution of hydrochloric acid, hydrofluoric acid, nitric acid, and perchloric acid, we used an atomic absorption spectrophotometer (ISSCAS 1978) to assess the heavy metals (HMs) (Cd, Zn, Fe, Cu, and Pb) in the soils.

### DNA extraction, PCR, and Illumina sequencing

Following the manufacturer’s recommendations, the Fast DNA SPIN Extraction Kit (MP Biomedicals, Santa Ana, CA, USA) was used to extract total AMF DNA from soil samples that had been collected. A NanoDrop 2000 spectrophotometer (Thermo Scientific, Wilmington, USA) was used to determine the concentration and purity of the DNA, and 1% agarose gel electrophoresis was used to evaluate the quality and integrity of the DNA. Following quality control and purification, soil AMF-specific primers proposed by Beenhouwer (17), Dai (18), Geel (19), Liang (20), and Xiang (21) et al. were utilized to amplify the internal transcribed spacer (ITS) region of AMF in certified DNA samples. The primers utilized were AMV4-5NF (5′-AAGCTCGTAGTTGAATTTCG-3′) and AMDGR (5′-CCCAACTATCCCTATTAATCAT-3′), which were synthesized by Novogene Co., Ltd. In order to prepare the sequencing library, the amplified target segment was purified and recovered using the magnetic bead technique after the amplified PCR product was detected on a 2% (w/v) agarose gel.

Following standard procedures supplied by Majorbio Bio-Pharm Technology Co., Ltd. (Shanghai, China), the sequencing library was made using the TruSeq DNA Sample Preparation Kit and sequenced on an Illumina MiSeq platform (2 × 300 bp, Illumina, San Diego, USA). The QIIME software suite was used to process and evaluate raw sequence data. On the basis of 97% similarity, nonredundant sequences—aside from singletons—were grouped into operational taxonomic units (OTUs). In order to provide representative OTU sequences, chimeric and low-quality reads were eliminated prior to grouping. The Ribosomal Database Project (RDP) classifier (https://sourceforge.net/projects/rdp-classifier/) was used to classify and annotate the representative OTU sequences by comparing them to the UNITE_Version7 database.

### Analysis of the habitat generalists and specialists

Levins’ niche width approach was used to assess the specialization of AMF habitats (22). For this research, the "Niche.Width" function from the R package "Spaa" was specifically utilized. AMF communities were categorized into generalists or specialists based on Levins’ niche width. Using the permutation method of the EcolUtils package, we performed 1,000 random reshufflings of the occurrence frequencies of OTUs and calculated the null distribution of their niche width indices. OTUs with niche width indices above the 95% confidence interval (CI) upper limit were designated as “generalists,” while those below the lower limit were identified as “specialists” based on the simulated null distribution.

### Statistical analyses

The diversity index of AMF OTU was computed for every sample using the diversity function in the “vegan” package. Bray-Curtis dissimilarities were used to examine the composition of the AMF community, and the “metaMDS” function was used to depict the results using nonmetric multidimensional scaling (NMDS). Using multiple linear regression and the “lm” function, the link between HMs and distance was investigated. The Shapiro-Wilk test was used to determine whether the residuals were normal. The “VennDiagram” tool in R was used to create Venn diagrams that quantified the distinct and common OTUs at various urbanization levels.

The analysis was performed using the beta nearest taxon index (βNTI) to evaluate the relative importance of stochastic and deterministic processes in assembling the AMF community. Specifically, R v.4.0.5’s “picante” packages were used to compute the βNTI values. βNTI values between −2 and 2 indicate that stochastic mechanisms, such as drift, dispersion limitation, or homogenizing dispersal, predominate in the construction of microbial communities. Deterministic processes (e.g., variable or homogenous selection) are indicated by βNTI values <−2 or >2. The Nemerow composite index method is one of the most commonly employed techniques for calculating environmental composite pollution indices. Based on the single-factor pollution index approach, it incorporates both the average and maximum values of individual elemental pollution indices, thereby emphasizing the impact of the most severely polluting factors. An index value exceeding 1 indicates environmental contamination.

The neutral community model (NCM) was further used to fit the relationship between the nonlinear least squares method of each OTU to evaluate the effect of stochastic process on the AMF community assembly. “m” and “R2” stand for the AMF community’s immigration rate to the NCM and goodness of fit, respectively. The association between HMs and βNTI in various communities was investigated using Spearman rank correlation. Finally, model computations were performed with R v.4.0.5.

## RESULTS

### Soil edaphic conditions and HM concentrations of urban green spaces

The soil conditions and HM pollution levels of 60 soil samples were evaluated. The results showed that, compared to urban soils, rural soils had better quality with higher available phosphorus (AP), pH, soil water content (SWC) values, and HM levels, as well as lower soil organic matter (SOM). The differences in concentrations were significant for SOM along the urban-rural gradient (Fig. 1b; Fig. S1).

**Fig 2:**
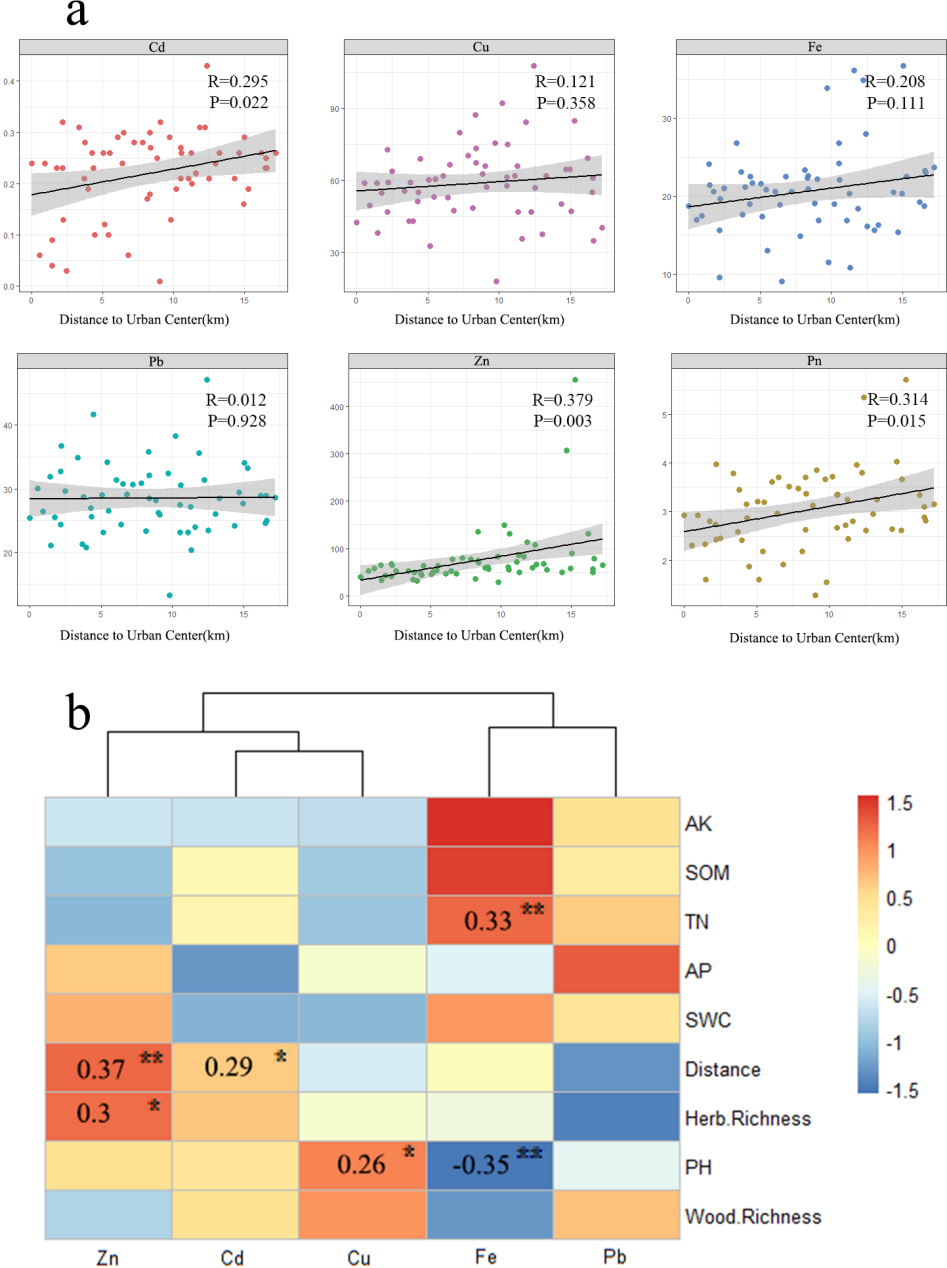
Relationships of HMs with the environmental factors. (**a**) Correlation between heavy metals and distance to urban centers and (**b**) correlations between heavy metals and soil conditions and plant abundance. ***P < 0.001, **P < 0.01, *P < 0.05.

The mean levels of all five HMs in the urban green soils of Zhengzhou were all higher than the background values of soils in Henan (Fig. 1). In particular, Cd and Zn increased with distance from the urban center, and Pn values also increased with distance (Fig. 2a). Fe was observed to be positively correlated with TN and negatively correlated with pH. The relationship between copper (Cu) and pH value was positively correlated (Fig. 2b).

### Compositional structures of urban green space AMF communities

**Fig 3:**
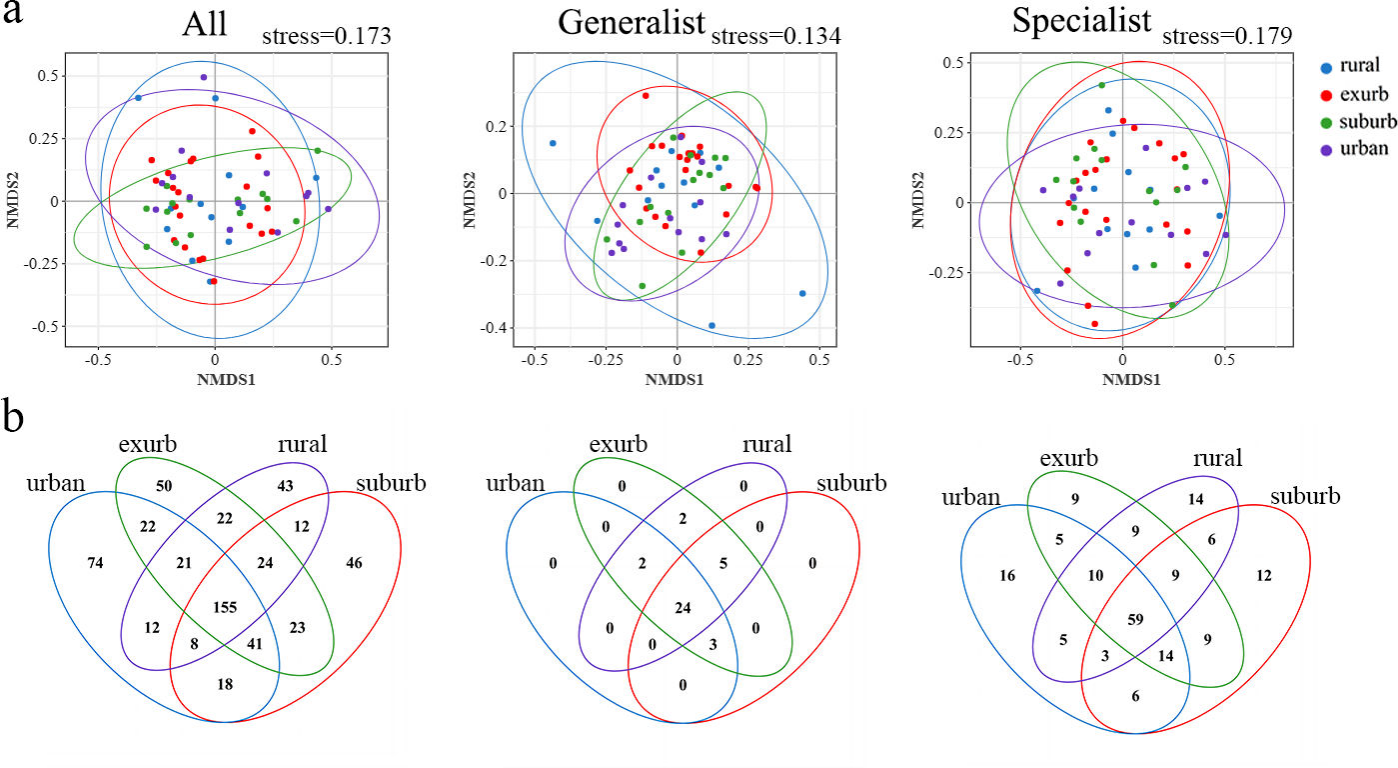
The composition of the microbial community. (**a**) Nonmetric multidimensional scaling (NMDS) plot for microbial communities across the four urban categories. (**b**) Venn diagrams were used to compare unique and shared taxa, specialists, and generalist taxa OTU across urban categories, respectively.

Based on the niche width index, 36 OTUs were identified as generalized species, while 186 OTUs were classified as specialized species. NMDS analysis of the AMF community assembly revealed that species composition varied among the three taxa across the four urbanization levels (Fig. 3). Generalized species shared a higher proportion of OTUs (66.67%) compared to specialized species and species across all urban categories (Fig. 3). The proportions of AMF at the genus and species levels varied along the urban-rural gradient. Notably, Glomus_f_Diversisporaceae was exclusive to rural areas. With increasing distances from the urban center, the proportion of all species, specialized species, and generalized species Glomus_f_Glomeraceae increased and gradually decreased, with the largest proportion found in the exurb areas. At the species level, Glomus-Glo16-VTX00120 was found only in rural areas. The unclassified_c_Glomeromycetes, Glomus-MO-G23-VTX00222, and unclassified_g_Diversispora accounted for relatively low proportions in the suburb and rural areas but relatively high proportions in the urban and exurb.

### Diversity of AMF communities

**Fig 4:**
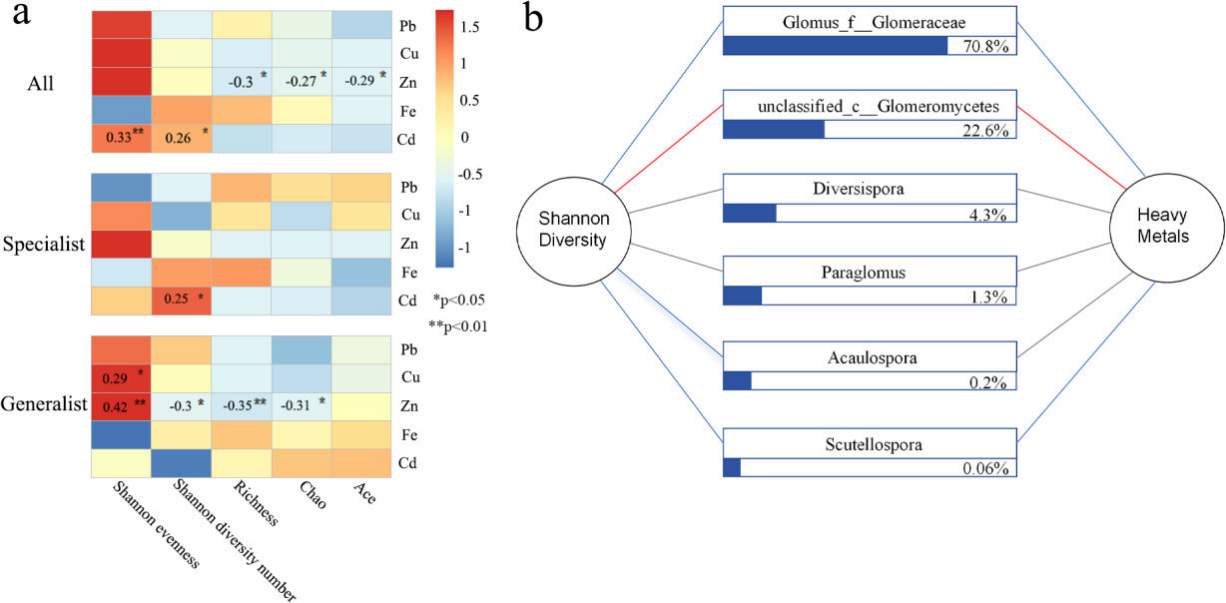
Alpha diversity indices of AMF communities. (**a**) The alpha diversity indices of all, generalist, and specialist communities, and their correlations with environmental factors. (**b**) Correlations between Shannon’s diversity and HM values for dominant classes. Positive and negative correlations are shown by blue and red lines, respectively. The relative abundance of each class is indicated within the rectangles.

The sparse curve analysis showed that richness plateaued as the number of detected sequences increased, indicating that most AMF sequences were captured and represented the majority of the information (Fig. S2). Diversity indices showed varying responses to HMs in AMF communities. In particular, Zn was positively correlated with AMF communities Shannon’s diversity but negatively correlated with richness indices, while Cd and Cu were positively correlated with the AMF communities (Fig. 4a). Mantel test results showed that only Zn was significantly associated with all three communities (*P* < 0.05) (Fig. S3).

Among the six classes, Acaulosporawith was positively correlated with Shannon’s diversity, but not with HMs. Unclassified_c_Glomeromycetes showed negative correlations with HMs and Shannon’s diversity. Scutellospora and Glomus_f_Glomeraceae exhibited positive correlations with both factors, while the other two genera showed no significant relationship (Fig. 4b).

### Assembly processes and driving factors for AMF communities

**Fig 5:**
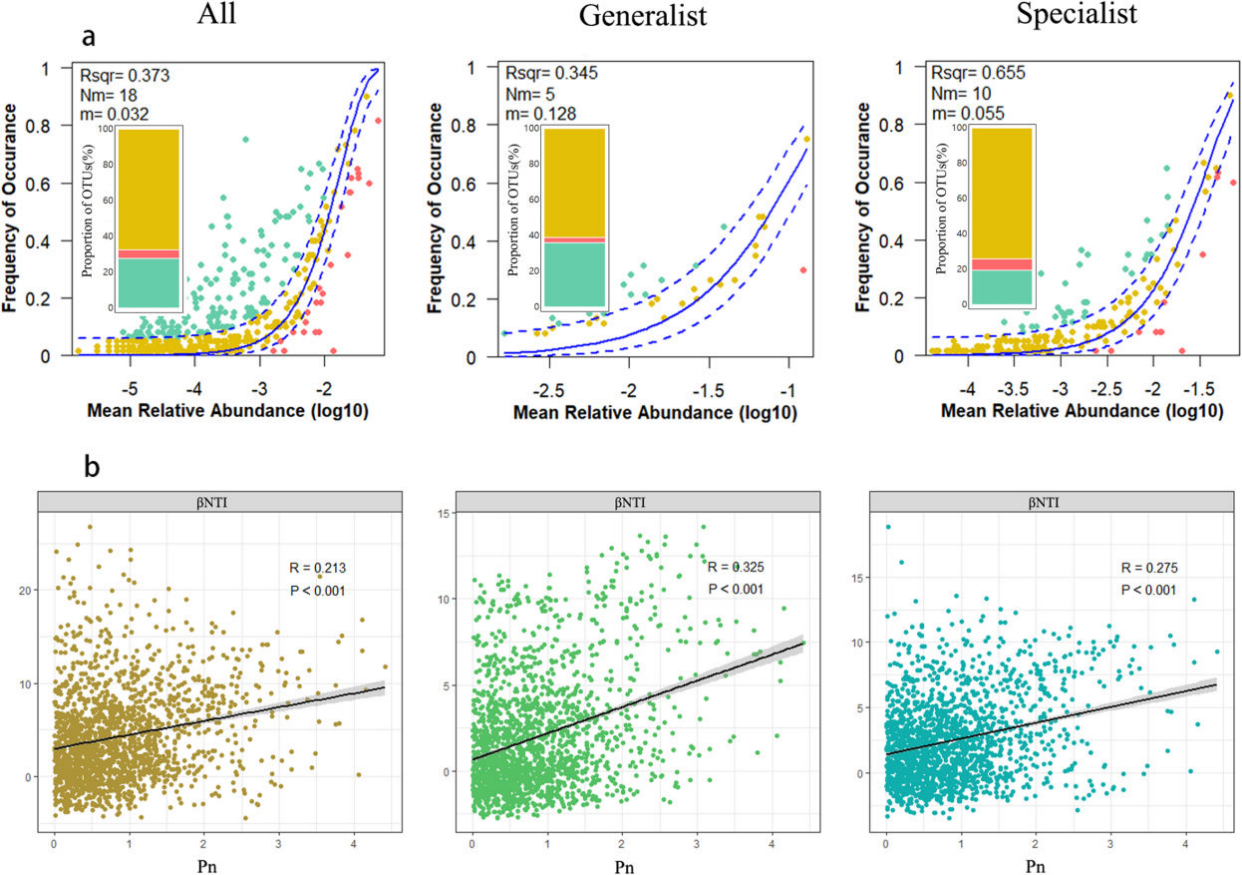
(**a**) Inference of the microbial community assembly process based on NCM. The blue solid line represents the best fit with NCM, and the blue dashed line indicates the 95% CI predicted by NCM. Dark green and red dots represent OTU occurrence frequencies that are higher and lower than those predicted by NCM, respectively. *R*² indicates the goodness of fit with NCM, and *m* represents the migration rate of the microbial community. (**b**) Linear regression analysis of the relationship between soil microbial community *β*-nearest taxon index (βNTI) and Pn (Nemerow composite pollution index). The gray-shaded area represents the 95% CI (*P* < 0.05); *P* < 0.05 means the linear relationship between βNTI and Pn is statistically significant, not random.

Our results showed that βNTI values were greater than two for all species and specialist species. In contrast, the βNTI values for generalist species ranged from −2 to 2. These results suggest that stochastic processes primarily drive the assembly of generalist species, while the assembly of all species and specialist species was mainly dominated by deterministic processes. NCM effectively captured the relationship between OTU occurrence frequency and their relative abundance, with *R*² values explaining 37.3, 34.5, and 65.5% of the community variance for all taxa, generalist taxa, and specialist taxa, respectively (Fig. 5a). Additionally, 67.42, 61.11, and 74.2% of OTUs were predicted to follow a neutral distribution, with confidence levels of 95% for all species, generalists, and specialists, respectively. In addition, a higher *m* value was observed in generalist taxa (0.128) compared to all taxa (0.032) and to specialist taxa (0.055). This finding suggests that weaker immigration ability exists in all and specialist communities due to stronger dispersal limitation. As a result, stochastic processes have a lesser role in the assembly of all taxa and specialist taxa, suggesting that deterministic processes primarily drive community assembly. These findings align with the null model analysis results.

The results of the linear regression analysis showed that the change of Pn was significantly positively correlated with βNTI (Fig. 5b, *P* < 0.001); Fe was significantly negatively correlated with βNTI, while Zn showed a positive correlation with βNTI in three taxa (Fig. S5, *P* < 0.001). These findings indicate that HM contamination significantly shapes microbial community composition in the region, with specialized species primarily driven by stochastic processes.

## DISCUSSION

### Varied HM concentrations under different urbanization levels

In this study, we found that the concentrations of HMs and the physicochemical properties of soils differed based on varying levels of urbanization. The finding that SOM decreased with increasing distances from the urban center is similar to that of a previous study (23). The TN levels of the urban soil were greater than those in the suburb, exurb, and rural areas, probably due to the green application of nitrogen manure and animals, as well as the deposition of nitrogen-containing compounds in motor vehicle exhaust (24). The concentrations of all HMs markedly exceeded the background levels of Zhengzhou soils. In this study, the content of HMs (except Pb) and the comprehensive pollution indices typically decreased with the increasing distances from the urban center, contrary to the study of Li et al. (25). In agricultural soils, HMs can accumulate in poorly soluble forms, leach into the soil solution, and eventually harm groundwater and crop quality (26). The long-term repeated application of metal-containing pesticides, chemical fertilizers, and fungicides causes the gradual accumulation of HMs leading to potentially harmful levels in the soil (27). Therefore, in the context of urban green spaces, the concentrations of HM pollutants tend to be higher in suburban and rural areas than in urban areas.

According to the Spearman’s correlation analysis (Fig. 2b), significant correlations were found between HMs and several soil properties, such as TN and pH, which may result from soil substances inhibiting the transfer of HMs. Many studies have reported that soil structure, cation exchange capacity (CEC), and electrical conductivity also have essential effects on the migration of HMs in soil (28–31). Mazurek et al. (32) demonstrated that HMs can combine with organic matter to form solid compounds, becoming immobilized in the soil. Soil pH typically regulates the adsorptive interactions between HMs and sorbents, including the complexation of dissolved organic matter (DOM) with Cu, Cd, Zn, Ni, Pb, Cr, Fe, Sb, and As in soil solutions (33–35). The results suggest that TN and pH are important factors influencing the distribution of HMs in urban green space ecosystems.

### Effects of HMs on community structure and diversity of AMF

Soil AMF diversity analysis revealed a negative correlation between Zn and overall species richness, while Cd showed a positive correlation with the Shannon diversity index of specialist species (Fig. 3a). According to Moffett et al. (36), elevated Zn levels reduced bacterial community diversity in arable soils, while Macdonald et al. (37) reported that Zn pollution was an important factor affecting the microbial community structure. Cd contamination may decrease the diversity of generalist species in AMF communities while enhancing the diversity of specialist species. Unclassified fungi were most abundant in unpolluted sites, showing a negative correlation with soil Zn pollution. Therefore, the HMs Zn and Cd are important factors affecting the AMF diversity and community structure in the urban green space ecosystem.

The results of community analysis revealed that Glomeraceae were dominant fungi at the urbanization level, and Glomeraceae had the highest proportion in the exurb. After comparing the exurb with other areas, we found that the Cu content in the exurb was higher than that in other areas (Fig. 1b; Fig. S4). This may be due to the survival strategy of AMF in Cu-polluted environments. Cornein reported the presence of blue-green fungal spores in such environments and found that the proportion of blue-green spores in the fungal community increases with Cu pollution. Moreover, the spores with accumulated Cu are inactive, indicating one of the strategies for fungi to survive in copper-polluted environments (38). Surveys on AMF diversity across different habitats, including agricultural soils, often report the dominance of Glomus species (18), urban green soils, alpine wetlands (3), and tropical forest soils (39). Similar to our results, Krishnamoorthy et al. reported Glomus dominance in HM-contaminated sites. However, unlike other AMF species requiring spore germination, Glomus isolates can utilize root fragments and hyphae as effective propagules (40). Due to the strong tendency for anastomosis formation in Glomus strains, they are able to expand their extramatrical mycelial network, facilitating more efficient distribution and allocation of resources (41). This phenomenon may be the reason why the Glomus group emerged as the most dominant genus in our study.

### Influence of HMs on the community construction of generalists and specialists

The βNTI analysis indicated that stochastic processes played a larger role than deterministic processes in shaping the assembly of the generalist community within the urban green space ecosystem. This finding is consistent with previous studies, which found that the distribution of generalists was mainly influenced by neutral processes, as they are less affected by changes in habitat conditions. In contrast, species sorting (deterministic processes) has a greater influence on habitat specialists, as they are adapted to specific environmental conditions (12, 42).

Moreover, the NCM results indicated that stochastic processes play a more significant role than deterministic processes in the assembly of generalist communities. In contrast, deterministic processes were more important than stochastic processes in the assembly of specialist communities in urban greenspace ecosystems. Such differences may be attributed to habitat heterogeneity and environmental stress variations (43). In temperate urban green spaces, deterministic processes govern the community assembly of specialists, while stochastic processes drive the assembly of generalists.

Our findings further revealed a strong association between HM and the βNTI values for both generalists and specialists (Fig. 5; Fig. S5). This suggests that HM plays a key role in regulating the balance between stochastic and deterministic assembly processes for both generalists and specialists in urban green spaces. Previous studies have identified soil chemistry as a key factor affecting the assembly process of soil AMF communities in many different environments, such as pH, available sulfur, organic matter, and NH4+-N (44–47). The trade-off between generalists and specialists plays a significant role in determining the community’s potential to adapt to new environments. For the generalists, the stochastic assembly process can be seen as a means of acclimatization to potential future environmental alterations. This can be explained by the fact that HMs are important factors affecting the community construction of generalized species and specialized species in urban green space ecosystems.

### Conclusion

This study examined AMF community responses to edaphic factors, with an emphasis on HM contamination, and explored the ecological mechanisms underlying the microbial community structure. We found that the concentrations of HM pollutants in suburban and rural areas were often higher than those in urban areas, and that TN and pH were important factors affecting the distribution of HMs in urban green space ecosystems. Notably, generalist community assembly was primarily driven by stochastic processes, while deterministic processes exerted a greater influence on specialist communities. Furthermore, HMs play a crucial role in guiding AMF community assembly, especially for Zn and Fe. In conclusion, this study provides valuable insights into how AMF communities respond to stress environments, further enriching our knowledge of soil microbial ecology within urban green spaces.

## Data Availability

The original genetic data obtained from ITS sequencing are available in the NCBI Sequence Read Archive under accession number PRJNA1182523.

## SUPPLEMENTAL MATERIAL

Supplemental material aem.00171-26-s0001.docx Figures S1 to S5.

ASM does not own the copyrights to Supplemental Material that may be linked to, or accessed through, an article. The authors have granted ASM a non-exclusive, world-wide license to publish the Supplemental Material files. Please contact the corresponding author directly for reuse.
