# Supplementary material for: Coexistence process and driving factors of arbuscular mycorrhizal fungi in urban green soil under heavy metal stress
Source: Appl Environ Microbiol. 2026 Mar 10;92(4):e00171-26. doi: 10.1128/aem.00171-26 (PMC13101536; doi:10.1128/aem.00171-26)
Supplement: Supplemental material — Figures S1 to S5. [file aem.00171-26-s0001.docx]

Supplementary material


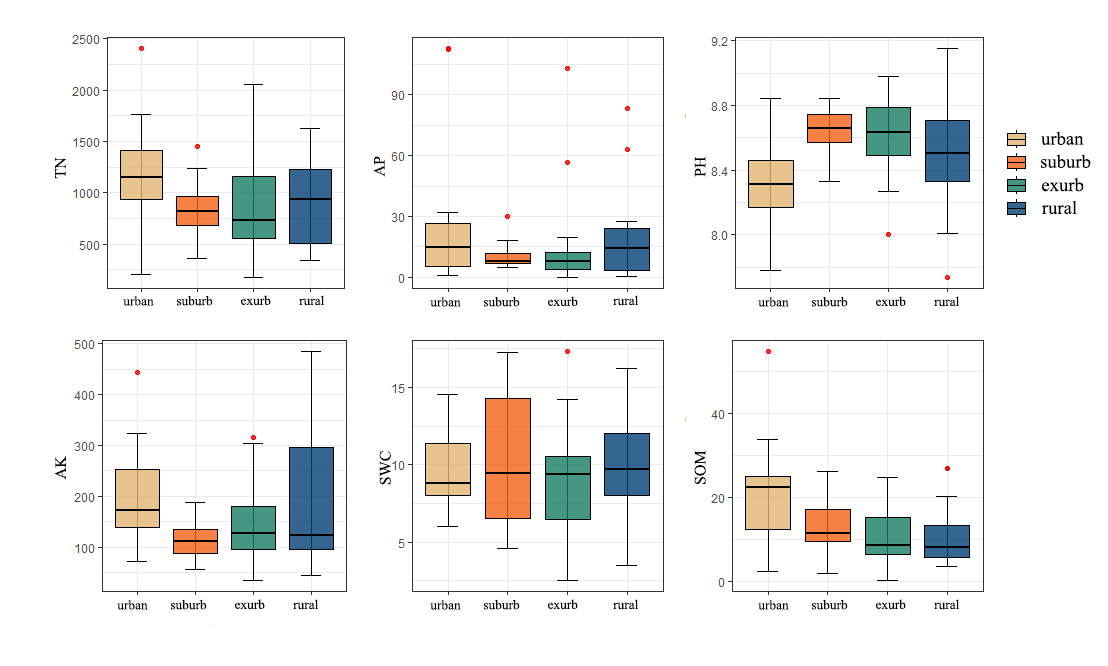


Fig. S1. Levels of soil characteristics across the four urban categories.


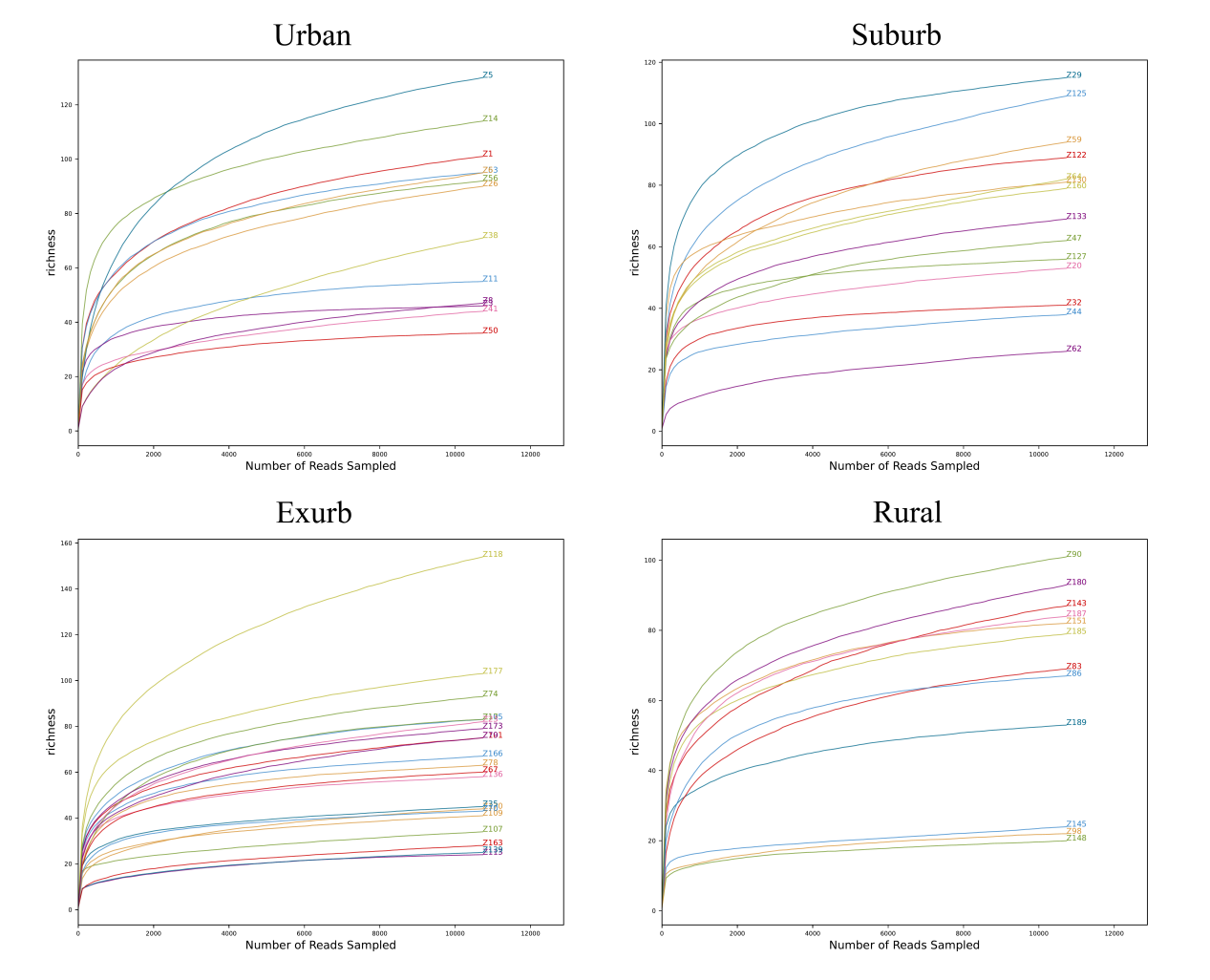


Fig S2 Sparse curves of AMF richness index under different urbanization levels.


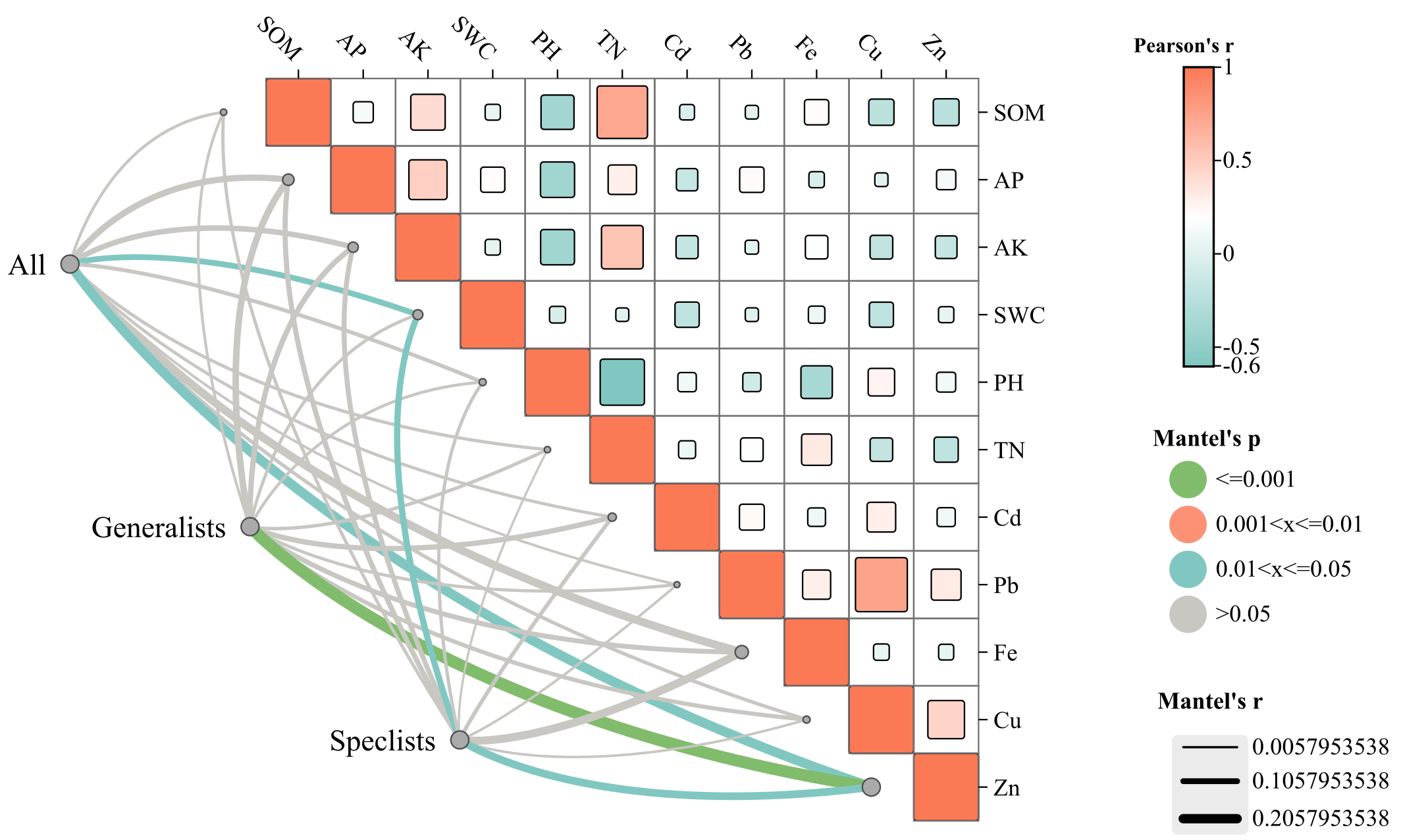
Fig. S3 Mantel Test of Relationships between Different Soil Fungal Communities and Soil Factors and Heavy Metal Content.


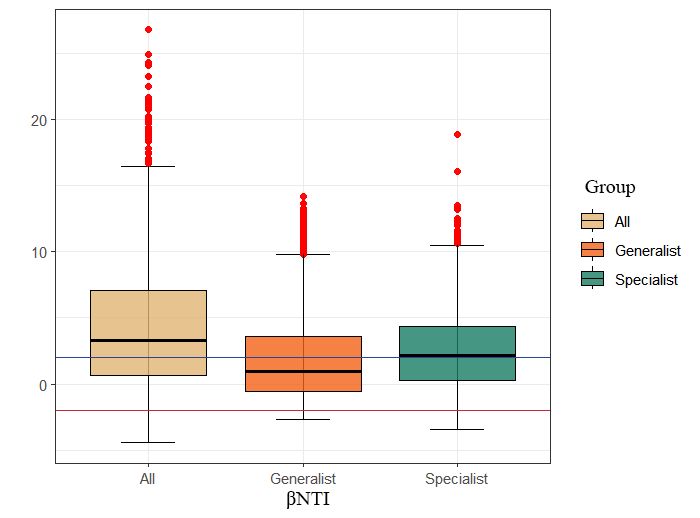


Fig. S4 Beta nearest-taxon index (βNTI) values of different AMF communities.


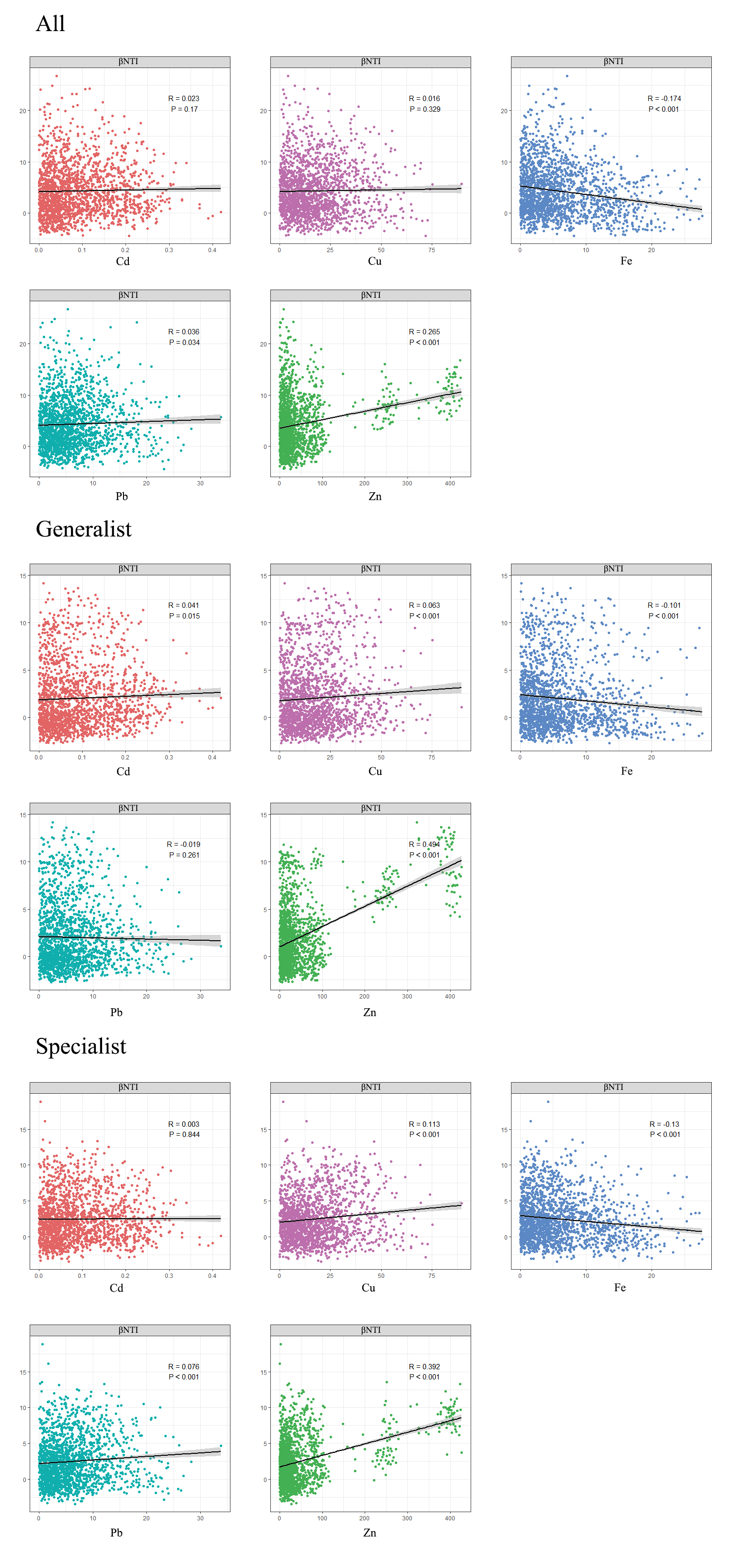


Fig.S5 Linear regression analysis of the correlation between βNTI values of soil fungal communities and heavy metal content.
